# Single-handed versus multiple-handed general practices: A cross-sectional study of quality outcomes in England

**DOI:** 10.1177/13558196231218830

**Published:** 2023-12-13

**Authors:** Ian Holdroyd, William Chadwick, Adam Harvey-Sullivan, Theodore Bartholomew, Efthalia Massou, Victoria Tzortziou Brown, John Ford

**Affiliations:** 1 Foundation Doctor, School of Clinical Medicine, 12204 University of Cambridge, Cambridge, UK; 2 Academic Clinical Fellow, Wolfson Institute for Population Health, 105714 Queen Mary University of London, London, UK; 3 GP Registrar, General Practice, 3661 Royal Surrey NHS Foundation Trust, Guildford, UK; 4 Research Associate, Department of Public Health and Primary Care, 2152 University of Cambridge, Cambridge, UK; 5 Senior Clinical Lecturer, Wolfson Institute for Population Health, 105714 Queen Mary University of London, London, UK; 6 Senior Clinical Lecturer in Health Equity, Wolfson Institute of Population Health, 105714 Queen Mary University of London, London, UK

**Keywords:** general practice, health care delivery, primary care, discipline: public health, health sector reform

## Abstract

**Objectives:**

As general practice increasingly moves towards large group practices, there is debate about the relative benefits, safety and sustainability of different care delivery models. This study investigates the performance of single-handed practices compared to practices with multiple doctors in England, UK.

**Methods:**

Practices in England with more than 1000 patients were included. Workforce data and a quality control process classified practices as single-handed or multiple-handed. Outcomes were (i) GP patient survey scores measuring access, continuity, confidence in health professional and overall satisfaction; (ii) reported diabetes and hypertension outcomes; and (iii) emergency department presentation rates and cancer detection (percentage of cancers diagnosed by a 2-week wait). Generalised linear models, controlling for patient and practice characteristics, compared outcomes in single and multiple-handed practices and assessed the effect of GP age in single-handed practices.

**Results:**

Single-handed practices were more commonly found in areas of high deprivation (41% compared to 20% of multiple-handed practices). Single-handed practices had higher patient-reported access, continuity and overall satisfaction but slightly lower diabetes management and cancer detection rates. Emergency department presentations were higher when controlling for patient characteristics in single-handed practices but not when also controlling for practice rurality and size. Increased deprivation was associated with lower performance in seven out of eight outcomes.

**Conclusions:**

We found single-handed practices to be associated with high patient satisfaction while performing slightly less well on selected clinical outcomes. Further research is required to better understand the association between practice size, including increasing multidisciplinary working, on patient experience and outcomes.

## Introduction

General practice is under considerable strain in the United Kingdom.^1–3^ In England, the proportion of patients reporting to have had a good overall experience fell from around 82% during 2018–2021 to 73% in 2022, a record low. Meanwhile, the number of those reporting poor experience doubled during the same period.^4^

In the United Kingdom, as in many other countries, GPs work from GP surgeries and act as a local population’s first point of contact for most health-related issues. In England, the average surgery list size increased from 6375 in 2006/7 to 9724 in April 2023.^5,6^ A 2016 national strategy seeking to strengthen primary care encouraged practices to collaborate and argued that ‘working at scale’ would increase access, innovation, staff development and voice and would also decrease costs and allow economies of scale to be achieved.^7^ Working at scale can take many different forms, such as more professionals working together in a larger physical space, GP surgeries delivering specific aspects of care at scale (e.g. anticipatory care being delivered by multiple GP practices working together) or physically distinct GP surgeries combining administratively and financially to various degrees.

Despite an overall trend towards larger group practices, a small number has remained ‘single-handed’, with one principal GP partner working as a single full-time GP. Other health care professionals may work at these practices. There is a weak, and dated, evidence base on the performance of single-handed practices within the English National Health Service (NHS). Using 2004–05 data, one study found that single-handed practices were associated with lower care quality as measured by points achieved under the national pay-for-performance scheme (Quality and Outcomes Framework, QOF) compared to a group practice,^8^ although access metrics did not differ. One other study used 2009–10 GP patient survey data and found that patients were more likely to leave practices with a small number of doctors than group practices, with single-handed practices having 2.75 times the disenrollment rate of practices with 6–9 doctors, suggesting higher levels of patient dissatisfaction.^9^

International evidence on the relative performance of single-handed compared to group practices points to the former having worse prescribing appropriateness and lower use of information technology.^10^ Evidence from the Netherlands suggests that single-handed practices perform better on patient-perceived service and accessibility, but worse on practice infrastructure measures such as premises, equipment and organisation.^11^ Another Dutch study reported that single-handed practice GPs worked longer hours and had a larger patient list size.^12^ One study from Germany found that patients in single-handed practices had 2.9 times greater odds of hospitalisation following acute respiratory tract infections.^13^

There is consensus in England that reform of general practice is vital, with leading policy experts supporting the idea of ‘working at scale’.^2,3^ This would result in an overall move away from existing single-handed GP practices in primary care. However, there is currently insufficient evidence to support this move.^10^ This study aimed to contribute to filling this evidence gap by investigating the quality of service offered by single-handed practices compared to practices with multiple GPs in England. A secondary aim was to explore the role of the age of GP in the performance of single-handed practices; this was against the background that single-handed practices will often only have one full-time doctor who will have been in a practice for a long time, and this might affect performance.^14^

## Methods

### Study design and data sources

We undertook a cross-sectional analysis. The reporting of this study conforms to the STROBE statement.^15^

We accessed NHS Digital^6^ to obtain (i) GP workforce data for 2020–2022, focusing on March and September each year to identify the total number of fully trained GPs (including part-time and temporary GPs), by age and sex, and the percentage of male and female patients in each practice; (ii) data on each practice’s contract type and rurality as of 2021–22 financial year; (iii) the number of patients at each practice as of January 2023; and (iv) QOF-reported diabetes and hypertension management for financial year 2021–22. The Office for Health Improvement and Disparities (OHID) fingertips API database^16^ was accessed to obtain data on deprivation (2019 Index of Multiple Deprivation (IMD) scores) and age profile for each practice, and on cancer detection and emergency presentation outcomes 2021–22. Finally, we used data on patient-reported experience from the 2022 GP Patient Survey.^4^ Practice data between datasets were linked by practice code. The dates of practice and patient data were chosen to reflect the time that outcomes were measured, choosing the most recent available data whenever possible.

#### Practice eligibility criteria

We excluded practices with under 1000 patients and those with no workforce data. Practices with alternative provider medical services (APMS) contracts were excluded as these tend to be atypical. AMPS contracts can be agreed with non-general practice organisations, such as in the private or third sector, and can be used to commission services beyond standard general practice care.

#### Definitions

NHS workforce or equivalent data commonly define a single-handed practice as one which has a single GP at a single point in time.^8,9,17^ However, this definition ignores practices for which the registration of only one GP may be transient due to a change in staff, or for accountancy reasons. It also includes GP practices working in a syndicate (whereby multiple practices join together), which are not single-handed GPs in the traditional sense. Also, GPs who work across multiple sites may not be registered to their main site of work. Including full-time GPs only ignores the work that locum (temporary) GPs perform.

We therefore defined a single-handed practice as a practice that had reported no more than one GP for at least 2 years before September 2022. This definition excludes practices operating within a syndicate and practices with more than 4000 patients as they were assumed to be too large to be feasibly single-handed.

#### Workforce variables

Each GP practice was labelled with a binary variable as to whether they were a single-handed practice. A data quality check was undertaken via manual retrieval of each practice’s website: any practice reporting more than one doctor on their staff pages, or with evidence that they were part of a syndicate with other practices was classed as multiple-handed. Practices which reported only one GP but had more than 4000 patients were labelled as multiple-handed as it is very unlikely that such a large number of patients could be managed by one GP alone, and workforce data was likely to be inaccurate. GP age was recorded as a categorical variable in each of the single-handed practices. Categories were under 50, 50–59, 60–69 and over 69 years.

#### Patient and practice variables

We used three variables to describe patient characteristics: patient age measured by the proportion of patients aged over 65 years in September 2022; patient sex measured by the proportion of patients who were male in September 2022; and deprivation using the 2019 IMD score for each practice. Practice characteristics included rurality defined as whether the practice was found in an urban or rural location; and the total number of patients defined as the number of patients reported as of 1 January 2023.

#### Outcomes

We considered a total of eight outcome measures, which are described in Table 1.

**Table 1.** Outcome variables.

Outcome (data source)	Data
Access (GPPS)	‘Overall, how would you describe your experience of making an appointment’? (very good; fairly good, neither good nor poor; fairly poor; very poor)
	Percentage of patients reporting ‘very good’ or ‘fairly good’
Confidence (GPPS)	‘During your last general practice appointment, did you have confidence and trust in the health care professional you saw or spoke to’? (yes, definitely; yes, to some extent; no, not at all; Don’t know)
	Percentage who reported ‘yes, definitely’ or ‘yes, to some extent’, excluding those who reported ‘Don’t know’
Continuity (GPPS)	‘How often do you see or speak to your preferred GP when you would like to’? (almost or almost always; a lot of the time; some of the time; never or almost never; I have not tried)
	Percentage who reported ‘almost or almost always’ or ‘A lot of the time’ excluding those who reported ‘I have not tried’
Overall satisfaction (GPPS)	‘Overall, how would you describe your experience of your GP practice’? (very good; fairly good; neither good nor poor; fairly poor; very poor)
	Percentage of patients reporting ‘very good’ or ‘fairly good’
Hypertension management (QOF)	The percentage of patients aged 79 years or under, with hypertension, in whom the last blood pressure reading (measured in the preceding 12 months) is 140/90 mmHg or less
Diabetes management (QOF)	The percentage of patients with diabetes, on the register, without moderate or severe frailty in whom the last IFCC-HbA1c is 58 mmol/mol or less in the preceding 12 months
Cancer detection (OHID fingertips)	Percentage of all new cancer cases treated resulting from a two-week wait referral pathway
Emergency department presentations (OHID fingertips)	Number of emergency presentations per 100,000 patients from a GP practice’s register

Note: Eight outcome variables utilised in analysis. GPPS: general practice patient survey; QOF: quality and outcomes framework.

### Statistical analysis and reporting

We compared single-handed and multiple-handed practices utilising three sets of generalised linear models for each outcome: (1) unadjusted; (2) controlling for patient characteristics (practice-level age, sex and deprivation score); and (3) controlling for patient characteristics and practice characteristics (practice rurality and total numbers of patients). Coefficients and standard errors were used to calculate the 95% confidence intervals shown in the figures. To ensure that when classifying practices with more than 4000 patients as multiple-handed, results were not affected by the cut-off size used, sensitivity analyses were ran utilising lower (3000 patients) and higher (5000 patients) cut-offs.

The effect of GP age in each of the single-handed practices was investigated utilising generalised linear models using the four GP categorical age groups. Models controlled for patient characteristics and total numbers of patients. Practice rurality was not included in the models due to a lack of rural single-handed practices.

A significant result was defined as *p* < .05. All analyses were conducted in R.

## Results

### Selection criteria and respondent characteristics

**Figure 1.**
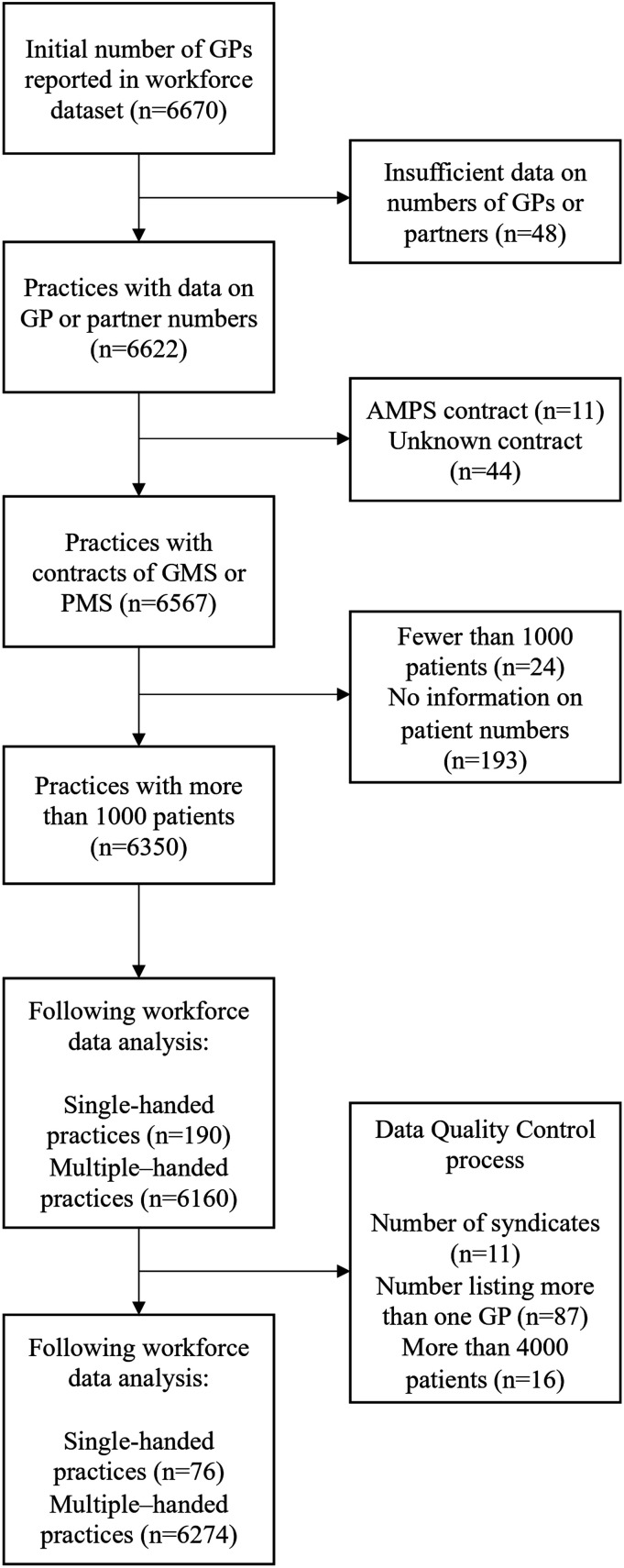
Selection criteria flowchart.

Of the 6670 practices in the workforce dataset, 6350 were included in analysis (Figure 1). The data quality check identified 114 practices that were not single-handed, or unlikely to be single-handed due to their size.

Table 2 shows included participants’ unweighted baseline descriptive statistics. Compared to multiple-handed practices, single-handed practices had lower mean weighted payments per patient, a higher proportion of male patients and a smaller proportion of patients over the age of 65. They were also more likely to be found in urban and deprived areas.

**Table 2.** Practice characteristics.

	Whole dataset	Single-handed practices	Multiple-handed practices
Total number of practices *N*	6350	76	6274
Contract type *N* (%)			
General medical services	4500 (70.9%)	55 (72.4%)	4445 (70.8%)
Personal medical services	1850 (29.1%)	21 (27.6%)	1829 (29.2%)
Weighted payment per patient mean (SD)	163.2 (46.7)	157 (51.4)	163.3 (46.7)
Mean practice list size mean (SD)	9774.5 (6599.4)	2655 (695.4)	9860.8 (6591.8)
Percentage male patients mean (SD)	50.3 (2.4)	52.7 (3.1)	50.3 (2.4)
Percentage of patients over 65 mean (SD)	17.5 (7)	16 (5.2)	17.5 (7)
Practice location *N* (%)			
Urban	5226 (82.3%)	68 (89.5%)	5158 (82.2%)
Rural	1052 (16.6%)	7 (9.2%)	1045 (16.7%)
Missing	72 (1.1%)	1 (1.3%)	71 (1.1%)
Deprivation *N* (%)			
1^st^ quintile (most deprived)	1268 (20%)	31 (40.8%)	1237 (19.7%)
2^nd^ quintile	1268 (20%)	14 (18.4%)	1254 (20%)
3^rd^ quintile	1269 (20%)	14 (18.4%)	1255 (20%)
4^th^ quintile	1269 (20%)	10 (13.2%)	1259 (20.1%)
5^th^ quintile (least deprived)	1269 (20%)	7 (9.2%)	1262 (20.1%)
NA	7 (0.1%)	0 (0%)	7 (0.1%)
Outcomes mean (SD)			
Access	58.5 (16.2)	68.9 (16.3)	58.4 (16.1)
Confidence	92.8 (5.2)	91.1 (6.4)	92.8 (5.2)
Continuity	38.8 (17.5)	66.1 (15.3)	38.7 (17.4)
Overall satisfaction	73.5 (13.1)	77.5 (12.1)	73.4 (13.1)
Hypertension management	57.8 (11.3)	57 (17.4)	57.8 (11.2)
Diabetes management	51.6 (7.4)	48.1 (10.3)	51.6 (7.4)
Cancer detection	53.3 (11.9)	48.3 (14.6)	53.4 (11.9)
Emergency department presentations	90 (48.4)	99.4 (66.7)	89.9 (48.1)

### Effect of a practice being single-handed

Table 3 shows regression analysis results comparing single-handed and multiple-handed practices. Results show effect sizes; beta values are the predicted difference caused by a practice being single-handed.

**Table 3.** Effect of a practice being single-handed.

	Model 1 - unweighted (ß and 95% CI)	Model 2 - controlling for patient characteristics (ß and 95% CI)	Model 3 - controlling for patient and practice characteristics (ß and 95% CI)
Access (GPPS)	10.50 (6.84, 14.10)	11.00 (7.45, 14.50)	8.10 (4.64, 11.60)
Confidence (GPPS)	−1.71 (−2.90, −0.53)	−0.29 (−1.36, 0.78)	−0.14 (−1.22, 0.95)
Continuity (GPPS)	27.40 (22.10, 32.80)	28.20 (22.9, 33.4)	23.9 (18.9, 28.9)
Overall satisfaction (GPPS)	4.05 (1.08, 7.01)	6.09 (3.29, 8.88)	4.59 (1.80, 7.38)
Hypertension management (QOF)	−0.81 (−3.36, 1.74)	−1.11 (−3.68, 1.46)	−1.55 (−4.14, 1.03)
Diabetes management (QOF)	−3.55 (−5.23, −1.87)	−2.54 (−4.17, −0.92)	−3.03 (−4.66, −1.40)
Cancer detection (OHID fingertips)	−5.12 (−7.81, −2.43)	−3.48 (−6.10, −0.85)	−3.44 (−6.09, −0.80)
Emergency department presentations (OHID fingertips)	9.45 (−1.48, 20.4)	9.53 (0.43, 18.60)	7.88 (−1.31, 17.10)

Note: Model 1 = unweighted. Model 2 = controlling for patient characteristics (age, sex, IMD). Model 3 = controlling for patient characteristics (age, sex, IMD) and practice characteristics (practice rurality and total patient numbers). Beta coefficients shown show the effect of a practice being single-handed compared to multiple-handed.

In the unweighted model, single-handed practices had better patient experience of access, continuity and overall satisfaction. They performed worse for patient confidence in the health care professional they saw, QOF-reported diabetes management and cancer detection.

When controlling for patient characteristics (age, sex and deprivation), access, continuity and overall satisfaction remained higher in single-handed practices. Cancer detection and diabetes management remained worse. There was no difference in patient confidence, and emergency department presentations were higher in single-handed practices.

When both patient and practice characteristics were added to the model, perceived access was higher in single-handed practices by 8.1%, continuity by 23.9% and overall satisfaction by 4.6%. QOF-reported diabetes management was 3.0% lower and cancer detection was 3.4% lower. There was a statistically nonsignificant increase in emergency department presentations of 7.9 patients per 100,000 in single-handed practices.

Full model outputs with results for all covariates are shown in Online Supplement Tables S1-S5. When controlling for single-handed status, patient characteristics and practice characteristics, patient age was a significant predictor for access, confidence, overall satisfaction, QOF-reported diabetes management, cancer detection and emergency department presentations. Patient sex was predictive for access, confidence, continuity, overall satisfaction, QOF-reported diabetes management and cancer detection. A practice being in an urban area was associated in worse results in all eight outcomes. Larger patient list size was associated with lower access, continuity, overall satisfaction, QOF-reported diabetes and hypertension management and emergency presentations. Higher levels of deprivation were associated with lower access, confidence, continuity, overall satisfaction, QOF-reported diabetes management, cancer detection and emergency presentations. A 10-point increase in an area’s deprivation score resulted in a 1.5% decrease in QOF-reported hypertension management, a 1.3% decrease in cancer detection and 9.4 higher number of emergency department presentations per 100,000 patients.

Sensitivity analyses confirmed that results were robust to changes in practice size cut-off used in the data quality control process (Tables S6 and S7), with one exception: if a cut-off size of 5000 patients was used to classify likely multiple-handed practices, results showed significantly increased emergency department presentation rates in single-handed compared to multiple-handed practices. No changes were seen when a smaller cut-off size was utilised.

### Effect of GP age

Table 4 shows the results of regression analyses investigating the effects of single-handed GP characteristics. Of the 76 single-handed practice GPs, 13 were between 30 and 49, 25 were between 50 and 59, 19 were 60 to 69 and 16 were older than 69. We did not find significant effects of GP age in the four outcomes reporting patient-reported experiences. QOF-reported hypertension and diabetes management were lower in single-handed practices with a GP aged over 69.

**Table 4.** The effect of the age of a single-handed GP on outcome variables.

	Access	Confidence	Overall satisfaction	Continuity	Hypertension management	Diabetes management	Cancer detection	Emergency department presentations
Aged under 50	Reference	Reference	Reference	Reference	Reference	Reference	Reference	Reference
Aged 50–59	6.2 (−4.94, 17.3)	−0.90 (−5.6, 3.8)	0.91 (−7.8, 9.61)	2.34 (−12, 16.7)	−11.5 (−24.2, 1.14)	−7.07 (−14.2, 0.01)	−4.25 (−14.1, 5.64)	23.8 (−24.3, 71.9)
Aged 60–69	10.8 (−0.59, 22.2)	−0.69 (−5.48, 4.11)	3.25 (−5.63, 12.1)	3.83 (−12.2, 19.8)	−12.7 (−25.6, 0.23)	−5.37 (−12.6, 1.86)	4.04 (−6.05, 14.1)	3.83 (−45.3, 52.9)
Aged over 69	10.4 (−2.44, 23.2)	−0.92 (−6.33, 4.48)	1.55 (−8.47, 11.6)	0.74 (−15.7, 17.2)	−17.3 (−31.9, −2.7)	−12.1 (−20.2, −3.91)	−1.6 (−13, 9.79)	25.3 (−30.1, 80.7)

*Note.* Results of regression analysis investigating the effect of the age of a single-handed GP on the eight outcome variables. Age was categorised into categorical variables of: under 50, 50–59, 60–69 and over 69. Models included all single-handed practices with data on GP age (*n* = 73). Models controlled for patient age, patient sex, IMD and total number of patients. All numbers correspond to the beta coefficient and respective 95% confidence intervals.

## Discussion

Our study found that, in England, single-handed practices were more likely to be found in deprived and urban areas than multiple-handed practices. Single-handed practices were associated with higher patient-reported access, continuity and overall satisfaction. For clinical outcomes, QOF-reported diabetes management and cancer detection rates were slightly worse in single-handed practices. Emergency department presentation rates were slightly higher when controlling for patient characteristics. There was a considerable effect of patient and practice characteristics on outcomes, especially an area’s deprivation score. We did not find differences in outcomes that were attributable to GP age, except for QOF-reported diabetes and hypertension management which were lower for GPs above the age of 69.

### Strengths and limitations

The specialised roles that smaller practices may be able to fulfil within a local community were not considered in this study. A qualitative analysis of single-handed practices would be required to understand these roles, rather than the quantitative analysis of all practices which was performed in this study.

Our results were robust to a wide set of covariates across multiple models and we used a wide range of outcomes, including a combination of patient-reported, practice-reported and NHS-reported data. As continuity was measured by the extent to which a patient saw their preferred GP, greater perceived continuity in single-handed practices was expected. QOF outcomes may not be representative of patient management as these rely on practice reporting, which, in turn, is affected by administrative capacity. Also, we used two QOF indicators that relate to the management of prevalent chronic conditions and thus offered a quantifiable estimate of patient outcomes. However, we did not consider outcomes reflecting the management of other conditions, such as mental health or respiratory disease.

Our analysis did not consider GP practices with fewer than 1000 patients as we considered these to be unlikely to be fully operational, be representative or reflective of wider trends because of atypical size. Practices with more than 4000 patients were assumed to be multiple-handed. This cut-off was chosen following consultation with three practising GPs and two academics. While we would expect single-handed practices to have larger list sizes per practising GP,^12^ it is very unlikely that such high patient numbers would be manageable by a single doctor given that the mean list size per doctor was 2248 in September 2022.^6^ Cut-offs were unlikely to affect findings given that practice size was controlled for in the final models and also that sensitivity analyses confirmed results were robust to changes in the cut-off. Due to the 2-year cut-off required for a surgery to be classed as single-handed, this analysis has excluded surgeries which have merged or split during this time. However, given the short time period, this should not have affected a large number of practices.

In addition to size cut-offs, other aspects of the data quality control process resulted in many practices being classed as multiple rather than single-handed. This prevented a large number of false positive classifications which has likely occurred in other studies.

### Comparison to previous research

As noted above, earlier studies that used QOF outcomes found no association between single-handed practices and access.^8^ Increased practice size was not linked to differences in reported access, although it was associated with lower continuity.^18^ In our study, patients at single-handed practices reported higher overall satisfaction but they did not differ in terms of confidence in the health care professional they saw. This was surprising, with one earlier study indicating that confidence in a GP was an important factor explaining overall patient satisfaction.^19^ It may be that patients now value other factors such as access and continuity of care, which were rated higher in single-handed practices, but this would need to be confirmed in further research. Previous work found that smaller practices performed better in patient experience of care compared to measures of clinical outcomes,^20^ a finding also reported in one study from the Netherlands.^11^ However, while of significant importance, patient-perceived outcomes should not be taken as a proxy for objectively measured outcomes and overall quality of care.^21^ Other work further suggests that increased continuity is linked to better outcomes including emergency admissions.^22^ Our study did not find evidence for this to be the case in this small subgroup of single-handed practices, whereby despite considerably higher continuity, emergency admissions were nonsignificantly increased. Our study did not confirm an increased probability of hospitalisation from single-handed practices that was reported elsewhere.^13^ This may be because the latter study focused on respiratory infections as a cause of admission rather than all illnesses.

Importantly, this research raises questions regarding datasets used by some of the earlier studies. Previous research has classified single-handed practices based on a cross-sectional assessment of the number of GPs working in a practice. Data quality control checks were not completed. Therefore, such studies would have as single-handed practices: practices working as part of a syndicate, practices with inaccurate workforce data, practices that transiently have a single GP but do not act as a single-handed practice and practices that are too large to realistically be a single-handed practice for any other reason.

### Implications for policy and research

A move to larger GP practices is occurring in many other countries such as New Zealand^23^ and Australia.^24^ Some aspects of scaling up could move general practice away from the single-handed model, without sufficient recent evidence assessing or examining how these practices perform. This research provides some evidence to inform future policies on this issue.

Our analysis revealed associations between single-handed practices and better patient-reported outcomes but slightly worse clinical outcomes. The reasons for these associations are unclear. Continuity, which has been shown to result in trusting relationships,^25^ and the halo effect^21^ may partly explain the better patient-reported outcomes in single-handed practices. In addition, the smaller teams and capacity of single-handed practices may mean that QOF-related targets become harder to record. Relational continuity with a single GP, while important for people with complex needs,^26^ may not be feasible for all patients currently. In England, alternative models increasingly include a patient seeing a wider range of individuals with expertise in their respective fields of health and social care, such as nurses, pharmacists, social prescribers (who provide personalised support by connecting people to locally available activities, groups and services that can support their social and emotional needs) and physiotherapists. Such a multidisciplinary primary care service will likely reduce relational continuity with a single clinician and may aid access,^27^ but the effects of such a model on health care costs, patient experience and quality of care remain unclear. In view of the move away from single-handed practices and towards increasingly larger teams, there is a need to better understand the drivers for this study’s observations so that future models of care can achieve both better clinical outcomes and patient experiences irrespective of their size.

GP practice funding in England is calculated by the Carr-Hill formula, which has been criticised for failing to address the extra pressures faced by practices in more deprived areas.^28^ The strong, negative effect of an area’s deprivation across a wide range of outcomes strengthens such arguments.

Our study also raises further research questions. Despite efforts to accurately categorise practices as single-handed, further work is needed to ensure such practices are correctly and consistently identified and coded in national databases. In view of the increasing multidisciplinary working, there is a need to identify ways of objectively measuring access and clinician and team-based continuity. The localised role of GP practices within their communities could be better understood by utilising qualitative research methods. Case studies may help to identify other gaps that cannot be addressed by a quantitative approach and strengthen subsequent policy recommendations. Such case studies could explore the impact of different GP models on GP retention and on the practice’s ability to respond to change.

## Supplemental Material

Supplemental Material - Single-handed versus multiple-handed general practices: A cross-sectional study of quality outcomes in England Supplemental Material for Single-handed versus multiple-handed general practices: A cross-sectional study of quality outcomes in England by Ian Holdroyd, William Chadwick, Adam Harvey-Sullivan, Theodore Bartholomew, Efthalia Massou, Victoria Tzortziou Brown and John Ford in Journal of Health Services Research & Policy.
