# Supplementary material for: Single-handed versus multiple-handed general practices: A cross-sectional study of quality outcomes in England
Source: J Health Serv Res Policy. 2023 Dec 13;29(3):201–9. doi: 10.1177/13558196231218830 (PMC11151703; doi:10.1177/13558196231218830)
Supplement: Supplemental Material - Single-handed versus multiple-handed general practices: A cross-sectional study of quality outcomes in England [file sj-pdf-1-hsr-10.1177_13558196231218830.pdf]

Online Supplement

Table S1: Effect of patient age: effect of mean age increasing by 1 year

	Model 1- Unweighted (ß and 95% CI)	Model 2- Controlling for patient characteristics (ß and 95% CI)	Model 3- Controlling for patient and practice characteristics (ß and 95% CI)
Access (GPPS)	NA	0.489 (0.423, 0.556)	0.277 (0.206, 0.348)
Confidence (GPPS)	NA	0.171 (0.151, 0.192)	0.159 (0.137, 0.181)
Continuity (GPPS)	NA	0.319 (0.244, 0.394)	0.0656 (-0.0126, 0.144)
Overall Satisfaction (GPPS)	NA	0.454 (0.402, 0.506)	0.342 (0.285, 0.398)
Hypertension management (QOF)	NA	0.0452 (-0.0029, 0.0934)	-0.0226 (-0.0752, 0.03)
Diabetes Management (QOF)	NA	0.0869 (0.0564, 0.117)	0.0359 (0.00261, 0.0691)
Cancer Detection	NA	0.212 (0.163, 0.261)	0.189 (0.135, 0.243)
Emergency Presentations (NHS Fingertips)	NA	4.29 (4.11, 4.46)	4.52 (4.33, 4.7)

Supplementary table 1 Results of regression analysis investigating the effect of a practice's mean patient age increasing by 1 year. Outcome = model dependant variable. Model 1 = unweighted. Model 2= controlling for patient characteristics (sex, IMD). Model 3= controlling for patient characteristics (age, sex, IMD) and practice characteristics (practice rurality and total patient numbers).

Table S2: Effect of patient sex: effect of 1% increase in patient sex being male

	Model 1- Unweighted (ß and 95% CI)	Model 2- Controlling for patient characteristics (ß and 95% CI)	Model 3- Controlling for patient and practice characteristics (ß and 95% CI)
Access (GPPS)	NA	0.585 (0.395, 0.774)	0.323 (0.136, 0.509)
Confidence (GPPS)	NA	-0.254 (-0.313, -0.195)	-0.258 (-0.317, -0.198)
Continuity (GPPS)	NA	0.707 (0.472, 0.942)	0.364 (0.138, 0.591)
Overall Satisfaction (GPPS)	NA	-0.154 (-0.304, -0.00387)	-0.293 (-0.443, -0.143)
Hypertension management (QOF)	NA	0.0713 (-0.0641, 0.207)	-0.00891 (-0.145, 0.127)
Diabetes Management (QOF)	NA	-0.0237 (-0.11, 0.0621)	-0.0869 (-0.173, -0.000703)
Cancer Detection	NA	-0.188 (-0.327, -0.0483)	-0.173 (-0.313, -0.032)
Emergency Presentations (NHS Fingertips)	NA	0.163 (-0.317, 0.642)	0.269 (-0.216, 0.754)

Supplementary table 2 Results of regression analysis investigating the effect of a practice's proportion of male patients increasing by 1%. Outcome = model dependant variable. Model 1 = unweighted. Model 2= controlling for patient characteristics (age, sex, IMD). Model 3= controlling for patient characteristics (age, sex, IMD) and practice characteristics (practice rurality and total patient numbers).

Table S3: Effect of Deprivation: effect of 10 point increase of IMD deprivation score

	Model 1- Unweighted (ß and 95% CI)	Model 2- Controlling for patient characteristics (ß and 95% CI)	Model 3- Controlling for patient and practice characteristics (ß and 95% CI)
Access (GPPS)	NA	-2.14 (-2.51, -1.77)	-2.37 (-2.74, -2)
Confidence (GPPS)	NA	-0.957 (-1.07, -0.844)	-0.899 (-1.01, -0.783)
Continuity (GPPS)	NA	-1.98 (-2.4, -1.56)	-2.39 (-2.8, -1.98)
Overall Satisfaction (GPPS)	NA	-1.76 (-2.05, -1.47)	-1.87 (-2.17, -1.57)
Hypertension management (QOF)	NA	0.329 (0.0602, 0.598)	0.251 (-0.0249, 0.528)
Diabetes Management (QOF)	NA	-1.44 (-1.61, -1.27)	-1.5 (-1.68, -1.33)
Cancer Detection	NA	-1.52 (-1.79, -1.24)	-1.34 (-1.62, -1.05)
Emergency Presentations (NHS Fingertips)	NA	9.98 (9.03, 10.9)	9.53 (8.55, 10.5)

Supplementary table 3 Results of regression analysis investigating the effect of a 10 point increase in IMD deprivation score. Outcome = model dependant variable. Model 1 = unweighted. Model 2= controlling for patient characteristics (age, sex, IMD). Model 3= controlling for patient characteristics (age, sex, IMD) and practice characteristics (practice rurality and total patient numbers).

Table S4: Effect of Urban area (compared to rural)

	Model 1- Unweighted (ß and 95% CI)	Model 2- Controlling for patient characteristics (ß and 95% CI)	Model 3- Controlling for patient and practice characteristics (ß and 95% CI)
Access (GPPS)	NA	NA	-5.48 (-6.63, -4.33)
Confidence (GPPS)	NA	NA	-0.676 (-1.04, -0.317)
Continuity (GPPS)	NA	NA	-5.88 (-7.14, -4.62)
Overall Satisfaction (GPPS)	NA	NA	-2.96 (-3.88, -2.03)
Hypertension management (QOF)	NA	NA	-1.77 (-2.62, -0.91)
Diabetes Management (QOF)	NA	NA	-1.27 (-1.82, -0.732)
Cancer Detection	NA	NA	-1.61 (-2.49, -0.729)
Emergency Presentations (NHS Fingertips)	NA	NA	9.4 (6.35, 12.4)

Supplementary table 4 Results of regression analysis investigating the effect of a practice being in an urban area compared to in a rural area. Outcome = model dependant variable. Model 1 = unweighted. Model 2= controlling for patient characteristics (age, sex, IMD). Model 3= controlling for patient characteristics (age, sex, IMD) and practice characteristics (practice rurality and total patient numbers).

Table S5: Effect of Total Numbers of Patients: effect of increasing total number of patients by 1000

	Model 1- Unweighted (ß and 95% CI)	Model 2- Controlling for patient characteristics (ß and 95% CI)	Model 3- Controlling for patient and practice characteristics (ß and 95% CI)
Access (GPPS)	NA	NA	-0.52 (-0.578, -0.461)
Confidence (GPPS)	NA	NA	0.0165 (-0.00182, 0.0348)
Continuity (GPPS)	NA	NA	-0.748 (-0.812, -0.684)
Overall Satisfaction (GPPS)	NA	NA	-0.279 (-0.326, -0.232)
Hypertension management (QOF)	NA	NA	-0.157 (-0.2, -0.113)
Diabetes Management (QOF)	NA	NA	-0.124 (-0.151, -0.0961)
Cancer Detection	NA	NA	0.0806 (0.0359, 0.125)
Emergency Presentations (NHS Fingertips)	NA	NA	-0.0803 (-0.235, 0.075)

Supplementary table 5 Results of regression analysis investigating the effect of a practice's total patient numbers increasing by 1000. Outcome = model dependant variable. Model 1 = unweighted. Model 2= controlling for patient characteristics (age, sex, IMD). Model 3= controlling for patient characteristics (age, sex, IMD) and practice characteristics (practice rurality and total patient numbers).

Table S6: Effect of being single-handed, using cut off size of 3000 patients

	Model 1- Unweighted (ß and 95% CI)	Model 2- Controlling for patient characteristics (ß and 95% CI)	Model 3- Controlling for patient and practice characteristics (ß and 95% CI)
Access (GPPS)	12.8 (8.56, 17.1)	13.3 (9.1, 17.4)	10.4 (6.36, 14.4)
Confidence (GPPS)	-1.43 (-2.82, -0.0383)	0.0903 (-1.17, 1.35)	0.232 (-1.03, 1.49)
Continuity (GPPS)	30.5 (23.5, 37.4)	31.7 (24.8, 38.5)	27.3 (20.7, 33.8)
Overall Satisfaction (GPPS)	5.61 (2.14, 9.09)	7.7 (4.43, 11)	6.18 (2.93, 9.42)
Hypertension management (QOF)	0.173 (-2.82, 3.16)	-0.171 (-3.18, 2.84)	-1.03 (-4.04, 1.97)
Diabetes Management (QOF)	-2.91 (-4.88, -0.935)	-1.77 (-3.68, 0.143)	-2.45 (-4.35, -0.547)
Cancer Detection	-5.94 (-9.1, -2.79)	-4.19 (-7.27, -1.11)	-3.66 (-6.74, -0.574)
Emergency Presentations (NHS Fingertips)	11 (-1.83, 23.8)	8.57 (-2.1, 19.2)	7.47 (-3.23, 18.2)

Supplementary table 6 Results of regression analysis investigating the effect of a practice being single-handed, when 3000 patients was utilised as the cut-off for when a practice would be defined as multiple-handed rather than single-handed. Outcome = model dependant variable. Model 1 = unweighted. Model 2= controlling for patient characteristics (age, sex, IMD). Model 3= controlling for patient characteristics (age, sex, IMD) and practice characteristics (practice rurality and total patient numbers).

Table S7: Effect of being single-handed, using cut off size of 5000 patients

	Model 1- Unweighted (ß and 95% CI)	Model 2- Controlling for patient characteristics (ß and 95% CI)	Model 3- Controlling for patient and practice characteristics (ß and 95% CI)
Access (GPPS)	9.25 (5.84, 12.7)	10 (6.69, 13.3)	6.98 (3.74, 10.2)
Confidence (GPPS)	-2.07 (-3.17, -0.963)	-0.677 (-1.68, 0.326)	-0.561 (-1.57, 0.452)
Continuity (GPPS)	25.6 (20.8, 30.4)	26.5 (21.8, 31.2)	22.1 (17.6, 26.5)
Overall Satisfaction (GPPS)	2.88 (0.105, 5.65)	4.96 (2.35, 7.57)	3.37 (0.762, 5.98)
Hypertension management (QOF)	-0.107 (-2.49, 2.28)	-0.373 (-2.77, 2.03)	-0.895 (-3.31, 1.52)
Diabetes Management (QOF)	-3.61 (-5.18, -2.04)	-2.58 (-4.1, -1.06)	-3.13 (-4.65, -1.6)
Cancer Detection	-4.42 (-6.93, -1.9)	-2.76 (-5.22, -0.307)	-2.71 (-5.19, -0.239)
Emergency Presentations (NHS Fingertips)	11.1 (0.884, 21.3)	11.9 (3.37, 20.4)	10.7 (2.1, 19.3)

Supplementary table 7: Results of regression analysis investigating the effect of a practice being single-handed, when 5000 patients was utilised as the cut-off for when a practice would be defined as multiple-handed rather than single-handed. Outcome = model dependant variable. Model 1 = unweighted. Model 2= controlling for patient characteristics (age, sex, IMD). Model 3= controlling for patient characteristics (age, sex, IMD) and practice characteristics (practice rurality and total patient numbers).
